# ZSF1 lean rats – How healthy are they?

**DOI:** 10.1002/ame2.70111

**Published:** 2025-12-19

**Authors:** Antje Schauer, Beatrice Vahle, Volker Adams, Axel Linke, Antje Augstein

**Affiliations:** ^1^ Department of Internal Medicine and Cardiology, Heart Center Dresden – Laboratory of Molecular and Experimental Cardiology Technische Universität Dresden Dresden Germany

**Keywords:** cardiovascular function, HFpEF, HFpEF rat model, skeletal muscle function, ZSF1 lean rat

## Abstract

ZSF1 lean rats are widely used as controls in cardiometabolic studies involving ZSF1 obese rats, which develop a cardiometabolic syndrome and diastolic dysfunction at a young age due to a double leptin receptor mutation (Lepr^cp^ and Lepr^fa^). Although, lean littermates show no overt signs of cardiometabolic disease or diastolic impact, they belong to one of three genotypic variants, two of which carry one of the mutant Lepr alleles and, thus, doubt has been raised about their suitability as healthy controls. We compared 32‐week‐old female ZSF1 lean and Wistar rats regarding physiological, myocardial, vascular, skeletal muscle, and mitochondrial characteristics. Lean rats showed lower body weight but increased heart, kidney, and skeletal muscle mass. Despite thicker ventricular walls, systolic and diastolic function were preserved. Hemodynamically measured contractility was higher as underpinned by a higher mitochondrial respiratory capacity of LV fibers. However, left ventricular filling pressure was elevated, accompanied by increased ventricular stiffness. Endothelial function was preserved, but smooth muscle responsiveness was reduced, indicated by impaired SNP‐induced relaxation. Passive vascular stiffness mediated by collagenous fibers was significantly higher in lean rats. Skeletal muscle function was mostly preserved, though maximal specific force of the EDL was reduced. Taken together, ZSF1 lean rats are physiologically different from Wistar rats as they display enlarged myocardial dimensions accompanied by increased blood pressure and an incipient diastolic and vascular stiffness. Therefore, our data indicate an early phase of passive compliance disorder in ZSF1 lean animals, which might become more pronounced at an advanced age.

## Graphical abstract

This study aimed to examine the health status, specifically the physiology and myocardial, vascular, and skeletal muscle function of lean ZSF1 rats by comparing them to age‐ and sex‐matched Wistar rats. Compared to Wistar controls, ZSF1 lean rats exhibited lower body weight, but showed increased heart, and skeletal muscle mass. Cardiac systolic and diastolic function were preserved, though left ventricular filling pressure and myocardial stiffness were elevated. Hemodynamic measurements revealed enhanced left ventricular filling and mean arterial pressure. While endothelial function was unaltered, vascular smooth muscle responsiveness was reduced, as indicated by impaired SNP‐induced relaxation and increased passive vascular stiffness. Although maximal specific force of the EDL muscle was reduced, skeletal muscle function was largely preserved.

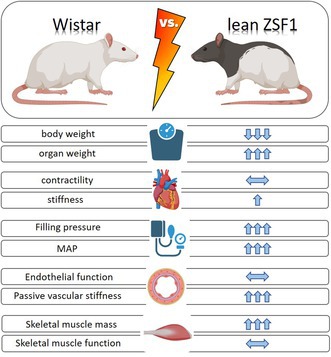

## 1 INTRODUCTION

Heart failure with preserved ejection fraction (HFpEF) remains a challenging and incompletely understood multifaceted disease with limited therapeutic options.^1^ As research depends on animal models reliably reflecting the clinical picture, the ZSF1 (Zucker fatty/Spontaneously hypertensive heart failure F1 hybrid) obese rat, carrying a double leptin receptor mutation (Lepr^cp^ and Lepr^fa^), has been shown to be superior to most other HFpEF models.^2^, ^3^ However, ZSF1 based research varies in the choice of appropriate control groups. Although the lean littermate does not develop obvious signs of cardiovascular dysfunction, it belongs to one of three possible genotypes (wt/wt, wt/Lepr^cp^ or wt/Lepr^fa^), harboring one of the parental mutant alleles with a 2/3 probability. Due in part to this heterogeneous genotype, concerns have been raised about its actual health status.^4^, ^5^, ^6^ In a recent study we demonstrated that relevant parameters of HFpEF, including left ventricular ejection fraction (LVEF), the ratio of early mitral inflow velocity to early diastolic mitral annular velocity (E/é) and N‐terminal pro‐B‐type natriuretic peptide (NT‐proBNP) levels are independent of the actual genotype.^6^ However, cardiomyocyte hypertrophy^7^ and enhanced blood pressure levels^8^, ^9^ were reported for lean ZSF1 rats and have not only reduced confidence in them as reliable controls but also raised the need to carry additional control groups.^9^ This study aimed to compare myocardial, vascular and skeletal muscle features of lean ZSF1 and Wistar rats to answer the question about the actual health status and, thus, the suitability of the lean ZSF1 littermate as a reliable control.

## 2 METHODS

### 2.1 Animals

All experiments were performed with female lean ZSF1 rats and Wistar‐Han rats at the age of 32 weeks. Wistar‐Han rats were considered the most appropriate control strain because lean ZSF1 rats are F1 progeny derived from ZDF (Zucker Diabetic Fatty rat) and SHHF (Spontaneously Hypertensive Heart Failure) parental strains, both on a Wistar background.^10^, ^11^ Until study termination, all animals were housed in groups of 3–5 per cage under standard laboratory conditions (22 ± 2℃, 50%–60% humidity, 12‐h light/dark cycle) with free access to standard rat chow and tap water. All procedures were carried out in accordance with national and European ethical regulations (Directive 2010/63/EU).

### 2.2 Echocardiography

At 32 weeks of age non‐invasive transthoracic echocardiography was performed using a Vevo 3100 imaging system equipped with a 21 MHz transducer (FUJIFILM VisualSonics Inc., Amsterdam, Netherlands), as previously described.^12^ To assess systolic function, B‐mode and M‐mode images were acquired in the parasternal long‐axis and short‐axis views at the level of the papillary muscles. Diastolic function was evaluated in the apical four‐chamber view using pulsed‐wave Doppler and tissue Doppler imaging at the basal septal segment of the septal wall of the left ventricle. Functional parameters were analyzed using Vevo LAB software (version 5.10.0).

### 2.3 Invasive measurement of hemodynamic parameters

Prior to organ harvest, invasive left ventricular hemodynamics were measured using a Rat PV catheter (SPR‐838, ADInstruments Limited, Oxford, UK) in anesthetized but spontaneously breathing rats as described before.^13^ Pressure‐volume loops were recorded under baseline conditions and during transient occlusion of the inferior vena cava by external compression of the vessel to obtain load independent indexes of contractility and chamber stiffness. The obtained end‐systolic and end‐diastolic pressure‐volume relationships (ESPVR, EDPVR) were fitted to linear and exponential functions, respectively, with the slope Ees indicating contractility and the chamber stiffness constant *β* displaying the grade of diastolic compliance. To take account of the different heart sizes between the groups, the left ventricular wall volume (*V*_*w*_) was used as a normalization factor (*β* * *V*_*w*_ = *β*_*w*_) as reported before.^14^, ^15^ Data were recorded and analyzed with LabChart 8 software (ADInstruments Ltd., Oxford, UK).

### 2.4 Measurement of vascular function and arterial stiffness

Ex vivo measurement of vascular function and arterial stiffness was performed as described before.^13^, ^16^, ^17^, ^18^ In brief, left carotid artery was dissected free of surrounding adipose and connective tissue and harvested for measurements of intrinsic mechanical wall stiffness. Two rings of approximately 0.5 mm were used. Unloaded internal circumference, vessel wall thickness and vessel length were determined using microscopic images. Carotid rings were mounted onto 200‐μm pins in heated myograph chambers (Danish Myo Technology, Aarhus, Denmark) with calcium‐ and magnesium‐free PBS. After resting for at least 30 min, two cycles of prestretching to approximately 5 mN for 5 min with 5 min intermission time were performed. Pins were adjusted to the zero position. Then the ring diameter was increased until a minimal force of approx. 0.5 mN was achieved. The ring was stretched incrementally every 3 min, the distance increase should be 5% to 10% of unloaded internal circumference. Distance and developed force F were documented until force exceeded 400 mN or ring broke and used to calculate stress and strain, defined as follows: Strain (ε) = r − r_0_/r_0_; ε is defined as relative deformation to baseline state, r_0_ is radius of unloaded ring, r is the radius of the specific step. Circumferential stress σ_c_ = (F*r)/(2*l*h*r_0_); σ_c_ is defined as force F per cross‐sectional area (h is wall thickness, l is segment length). The slope of the stress–strain curve was used to determine the elastic modulus E.

### 2.5 Skeletal Muscle Function

Skeletal muscle function of the right soleus was assessed as described before.^19^ The right soleus was dissected and mounted vertically in a Krebs–Henseleit buffer‐filled organ bath between a hook and force transducer, with the output continuously recorded and digitized (1205A: Isolated Muscle System—Rat, Aurora Scientific Inc., Aurora, ON, Canada). Ex vivo muscle function was assessed by platinum electrodes stimulating the muscle with a supra‐maximal current (700 mA, 500 ms train duration, 0.25 ms pulse width) from a high‐power bipolar stimulator (701C; Aurora Scientific Inc., Aurora, ON, Canada). The muscle was set at an optimal length (Lo) equivalent to the maximal twitch force produced. A force‐frequency protocol was then performed at 1, 15, 30, 50, and 80 Hz, separated by 1‐min rest intervals. After a 5‐min period in which muscle length was measured using a digital micrometer, the muscle underwent a fatigue protocol over 5 min (40 Hz every 2 s with a 500‐ms train duration). The muscle was subsequently detached, trimmed free from fat and tendon, blotted dry on filter paper, and weighed. Muscle force (N) was normalized to muscle cross‐sectional area (cm^2^) by dividing muscle mass (g) by the product of optimal length (cm) and estimated muscle density (1.06 g/cm^3^), which allowed specific force (in N/cm^2^) to be calculated.^12^, ^13^, ^20^

### 2.6 Mitochondrial respiration

Assessment of mitochondrial respiratory capacity was performed in saponin‐skinned muscle fibers of the left ventricle and of the soleus as described before.^15^ Respiratory rates were determined by using a Clark electrode (Strathkelvin Instruments, Motherwell, UK) in an oxygraphic cell at 25℃ with continuous stirring. To prevent limitations in oxygen diffusion, oxygen concentration was increased to ~400 μmol/L by adding pure oxygen and was kept above 270 μmol/L throughout the experiment. Left ventricular muscle fibers were isolated in permeabilization solution (SolP) containing (in mmol/L): 2.77 CaK2EGTA, 7.23 K2EGTA, 6.56 MgCl_2_, 5.7 Na_2_ATP, 15 phosphocreatine (PCr), 20 taurine, 0.5 DTT, 50 K methane sulfonate, imidazole (pH 7.1) and incubated for 30 min in SolP with 50 μg/mL saponin. Permeabilized fibers were transferred to respiration solution (SolR) (in mmol/L: 20 taurine, 20 HEPES, 10 KH_2_PO_4_, 0.5 EGTA, 3 MgCl_2_, 0.11 sucrose, 60 K‐lactobionate (pH 7.4)) for 10 min to wash out adenine nucleotides and PCr. All steps were carried out at 4℃ with continuous stirring. Respiration rates of 1–5 mg of skinned fibers were measured at 25℃ in 1 mL of SolR containing 1 mg/mL bovine serum albumin. The following substrates were added sequentially and oxygen consumption was monitored: (I) glutamate (10 mmol/L), malate (2.0 mmol/L), (complex I state 2 respiration); (II) adenosine diphosphate (5.0 mmol/L; measure of complex I oxidative phosphorylation); (III) octanoylcarnitine (0.2 mmol/L; measure complex I activated by fatty acid oxidation); (IV) cytochrome C (10 μmol/L; test for membrane integrity); (V) succinate (10 mmol/L; oxidative phosphorylation of complex I + II); (VI) rotenone (0.5 mmol/L; oxidative phosphorylation of complex II); (VII) FCCP (0.5 μmol/L, maximal uncoupled complex II respiration); (VIII) antimycin A (2.5 μmol/L, as a complex III inhibitor); (IX) ascorbate/N,N,N′,N′‐tetramethyl‐p‐phenylenediamine dichloride (2 mmol/L/0.5 mmol/L, maximal uncoupled complex IV respiration). Following the experiment, fiber bundles were blotted dry and weighed. Respiration rates are expressed as nmol O₂ per second per mg of wet tissue weight.

### 2.7 Data analysis

For vascular function analyses, statistical comparisons were made with two‐way ANOVA followed by Sidak's multiple comparison test. For all other analyses lean ZSF1 and Wistar rats were compared by two‐sided Student's *t* test of equal variances. Data are expressed as mean ± SEM. *p* values below 0.05 were considered to be statistically significant.

## 3 RESULTS

Despite their lower body weights, lean rats showed significantly enhanced weights of heart, kidney and skeletal muscles (Table 1). The higher left ventricular weight (*p* < 0.001) was accompanied by enhanced thickness of the left ventricular posterior wall (LVPW, *p* < 0.01) and septum (*p* < 0.01). However, these structural differences affected neither systolic nor diastolic functionality, which were comparable between both groups. Hemodynamic measurements revealed enhanced systolic (LVESP, *p* < 0.001) and diastolic filling pressure (LVEDP, *p* = 0.06) in lean rats, although statistical significance was not reached in the latter case. This was accompanied by increased mean arterial pressure (MAP, *p* < 0.001) and an increased LV‐stiffness constant *β*_*w*_ (*p* < 0.01). The slightly better contractile function indicated by slope LV‐Ees was supported by a higher mitochondrial respiratory capacity measured in complex I, II and IV of myocardial fibers (Table 1).

**TABLE 1:** Animal characteristics at the age of 32 weeks retrieved from physiological and morphometric measurements, echocardiography, invasive hemodynamic and mitochondrial function measurements.

Parameter	Wistar (*n* = 11)	ZSF1 lean (*n* = 10)
Physiology		
Organs		
Body weight [g]	292 ± 4	270 ± 4^#^
Tibia length [TL, mm]	37.0 ± 0.2	35.4 ± 0.07^#^
Lung wet weight /TL [mg/mm]	10.6 ± 0.3	10.9 ± 0.2
Kidney weight / TL [mg/mm]	24.8 ± 0.5	28.3 ± 0.6^#^
TA/TL [mg/mm]	13.37 ± 0.33	15.87 ± 0.22^#^
EDL/TL [mg/mm]	3.69 ± 0.09	3.95 ± 0.06*
SOL/TL [mg/mm]	4.07 ± 0.09	4.28 ± 0.06 (*p* = 0.07)
Sol max. specific force [mN/cm^2^]	38.41 ± 0.95	38.02 ± 0.87
EDL max. specific force [mN/cm^2^]	51.03 ± 1.94	43.66 ± 0.62^§^
Carotide wall thickness h [mm]	0.09 ± 0.0	0.08 ± 0.01
Carotide internal circumference [mm]	2.13 ± 0.09	2.15 ± 0.05
Carotide internal circumference 10 kPa [mm]	2.97 ± 0.12	3.01 ± 0.15
Echocardiography		
LV mass [mg]	543.9 ± 19.8	732.7 ± 33.04^#^
LVEF [%]	81 ± 2	80 ± 1
LVFS [%]	31 ± 1	29 ± 2
LVSV [μL]	282 ± 12	327 ± 17*
CO [mL/min]	93 ± 4.1	109 ± 5.7*
LVEDV [μL]	348 ± 15	409 ± 20*
LVESV [μL]	65 ± 7	82 ± 6
E/é	19.1 ± 1	18.5 ± 0.7
E/A	1.3 ± 0.04	1.4 ± 0.07
LVPW; d [mm]	1.5 ± 0.04	1.8 ± 0.09^§^
Septum; d [mm]	1.4 ± 0.07	1.7 ± 0.09^§^
LVID; d [mm]	7.6 ± 0.1	7.1 ± 0.1^§^
Invasive hemodynamics		
Heart rate [bpm]	246 ± 8	228 ± 6 (*p* = 0.07)
LVEDP [mmHg]	6.1 ± 0.8	8.7 ± 1 (*p* = 0.06)
LVESP [mmHg]	77.5 ± 3.4	111.6 ± 4.8^#^
MAP in asc. Aorta [mmHg]	70.0 ± 3.4	94.0 ± 5.5^#^
LVEDV [μL]	361.9 ± 20.1	385.7 ± 21.3
LVESV [μL]	90.5 ± 11.9	143.6 ± 12.1^§^
SW [mmHg × μL]	26 515 ± 1041	29 590 ± 2194
dP/dt max [mmHg/s]	6809 ± 295	7123 ± 210
dP/dt min [mmHg/s]	−5465 ± 232	−6817 ± 183^#^
dV/dt max [μL/s]	7064 ± 624	6864 ± 478
dV/dt min [μL/s]	6488 ± 456	5279 ± 348*
Tau [ms]	18.1 ± 0.77	17.7 ± 0.46
Slope LV‐Ees [mmHg/μL]	0.13 ± 0.02	0.21 ± 0.02*
LV‐stiffness constant *β*_*w*_	0.24 ± 0.03	0.45 ± 0.09^§^
Mitochondrial respiratory capacity		
Myocardium		
Complex I [nmol/s/mg]	0.084 ± 0.003	0.115 ± 0.010*
Complex II [nmol/s/mg]	0.121 ± 0.008	0.148 ± 0.010 (*p* = 0.06)
Complex IV [nmol/s/mg]	0.209 ± 0.017	0.279 ± 0.027*
Soleus		
Complex I [nmol/s/mg]	0.045 ± 0.003	0.050 ± 0.005
Complex II [nmol/s/mg]	0.079 ± 0.003	0.085 ± 0.009
Complex IV [nmol/s/mg]	0.137 ± 0.006	0.154 ± 0.019

*Note*: All data are displayed as mean ± SEM.

Abbreviations: A, late mitral inflow; a', tissue Doppler mitral annulus velocity in late diastole; bpm, beats per minute; CO, cardiac output; dP/dt max, maximum derivative of change in systolic pressure over time; dP/dt min, minimum derivative of change in diastolic pressure over time; dV/dt max, maximum derivative of change in volume over time; dV/dt min, minimum derivative of change in volume over time; E, early mitral inflow; é, tissue Doppler mitral annulus velocity in early diastole; EDL, extensor digitorum longum; LV, left ventricle; LVEDP, left ventricular end‐diastolic pressure; LVEDV, left ventricular end‐diastolic volume; LVEF, left ventricular ejection fraction; LVESP, left ventricular end‐systolic pressure; LVESV, left ventricular end‐systolic volume; LVFS, left ventricular fractional shortening; LVID, left ventricular inner diameter; LVPW, left ventricular posterior wall; LVSV, left ventricular stroke volume; MAP, mean arterial pressure; slope LV‐Ees, slope of left ventricular end‐systolic elastance; SOL, soleus muscle; SW, stroke work; TA, tibialis anterior; Tau, time constant of left ventricular relaxation; TL, tibia length.

* *p* < 0.05;

^§^ *p* < 0.01;

^**#**^ *p* < 0.001.

**FIGURE 1:**
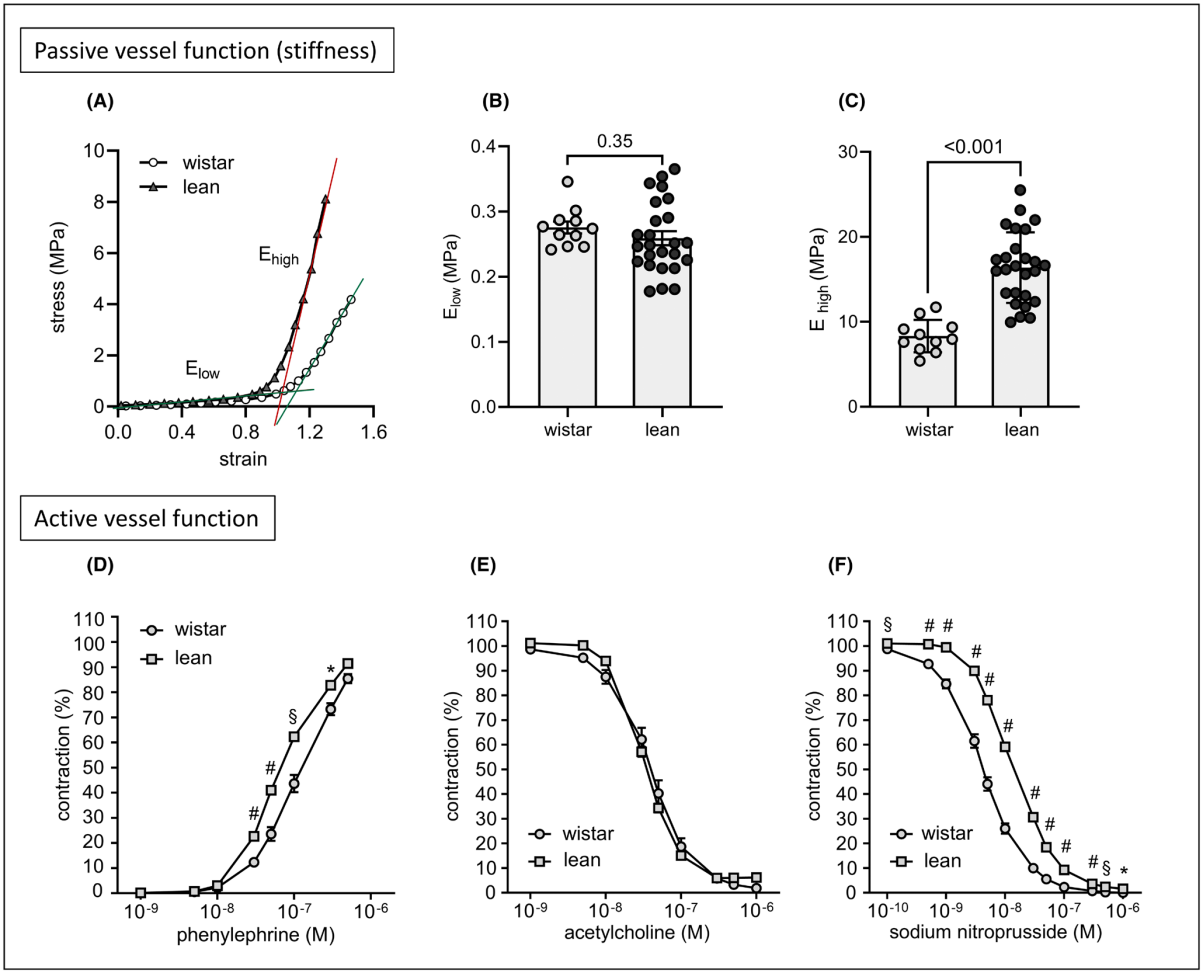
Stiffness and active vascular function of the left carotid artery in Wistar and ZSF1 lean rats. (A) Exemplary diagram of stress–strain curves for ex vivo measurements of carotid stiffness. Straight lines represent the slope used for estimation of elastic modulus E. (B and C), Slopes were calculated at low (E_low_) and high (E_high_) strains to differentiate between elastin‐ and collagen‐mediated stiffness, respectively. Statistical comparisons were made with unpaired *t* test. (D–F) Estimation of active vascular function using wire myography. (D) Contraction of carotids after phenylephrine administration. (E) Endothelial‐dependent relaxation after acetylcholine administration to preconstricted carotid rings. (F) Smooth muscle‐dependent relaxation after administration of sodium nitroprusside to preconstricted carotid rings. Statistical comparisons were made with two‐way ANOVA followed by Sidak's multiple comparison test. Wistar *n* = 11, ZSF1 lean *n* = 25. All data are displayed as mean ± SEM. *p < 0.05, ^§^ *p* < 0.01, ^#^ *p* < 0.001.

**FIGURE 2:**
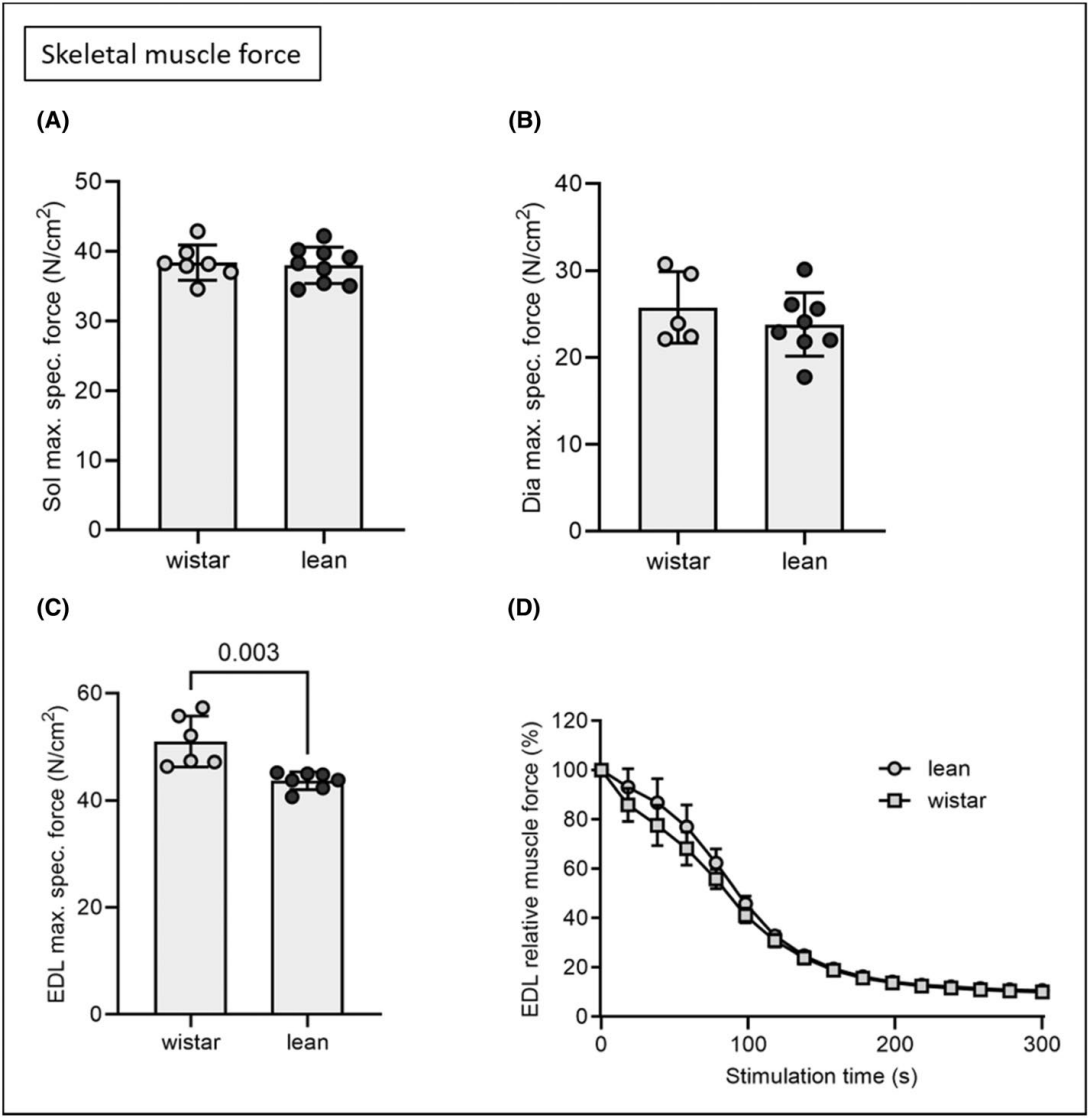
Maximal specific muscle force of soleus (Sol, A), diaphragm (Dia, B) and extensor digitorum longus (EDL, C). Muscle fatigue of the EDL muscle was assessed by repetitive stimuli (D). Lean ZSF1 and Wistar rats were compared by two‐sided Student's *t* test of equal variances. All data are displayed as mean ± SEM. Wistar *n* = 5–7, ZSF1 lean *n* = 7–9.

While carotid wall thickness and internal circumferences were comparable between both groups (Table 1), passive vessel function was highly impaired in lean rats, indicated by enhanced stiffness mediated by collagenous fibers (E_high_, Figure 1A–C). Also, lean carotid sections reacted more contractively in response to phenylephrine stimulation, possibly due to increased stiffness (Figure 1D). While the endothelial function was not different between both groups (Figure 1E), lean carotids were less responsive to sodium nitroprusside (Figure 1F), indicating an impaired relaxability of lean smooth muscle cells. The observed enhanced carotid stiffness positively correlated with the LVESP (*r* ^2^ = 0.527, *p* < 0.001). Other than a lower maximal specific force of the EDL in lean rats, no further differences were observed in skeletal muscle function (Figure 2A–D). This was confirmed by unaltered mitochondrial respiratory capacity of soleus fibers (Table 1).

## 4 DISCUSSION

While no functional impairment was observed, our data reveal a concentric left ventricular hypertrophy accompanied by an incipient diastolic and vascular stiffness in lean ZSF1 rats (Table 1, Figure 1). This might be the adaptive response to a hypertension‐driven increased afterload. The seemingly contradictory combination of elevated LVEDP and stiffness factor *β*_*W*_ but unaltered E/é ratio and Tau suggest an early phase of passive compliance disorder (Table 1). Moreover, the functional and structural alterations observed in carotids, namely the heightened response to α2‐agonist phenylephrine (Figure 1D) and the delayed donor‐mediated vasodilation (Figure 1F) were also reported by Leite et al.^21^ who compared aortic sections of lean ZSF1 and Wistar rats. However, in contrast to these finding, carotid wall thickness, and internal circumference remained unaltered, even when precontracted (Table 1). We speculate that the altered vascular structure observed in lean ZSF1 rats may either represent an adaptive response to elevated systemic pressure conditions or, conversely, that an intrinsically increased vascular stiffness—possibly of genetic origin—might be driving the development of hypertension. The “chicken‐or‐egg” question remains to be clarified in future work.

The observation that lean ZSF1 rats exhibit a heavier EDL muscle but a lower maximal specific force compared with Wistar rats (Table 1, Figure 2C) suggests that the increase in muscle mass is not proportionally translated into contractile capacity. This discrepancy is likely attributable to an expansion of non‐contractile elements, such as fibrosis or intramuscular fat, and/or a reduced density or altered isoform composition of contractile proteins within the myofiber cross‐sectional area.^12^, ^19^, ^22^

The observed cardiovascular alterations might be due to the fact that, statistically, two‐thirds of the lean offspring carry a parental mutation and both, Lepr^fa^ and Lepr^cp^, have been associated with metabolic and cardiovascular disorders.^23^, ^24^, ^25^, ^26^ Compared to Sprague–Dawley rats, male heterozygous ZDF rats showed increased levels of angiotensin I and mild‐to‐severe renal pathology at the age of 14 weeks, which might contribute to cardiac dysfunction at an advanced age due to the cardio‐renal connection.^24^ Regarding SHHF rats, data vary, with studies reporting ventricular hypertrophy present at 14 months of age^26^ and others demonstrating heart failure already at 10 months of age and several months earlier than in female rats.^25^ Although existing studies are mostly performed with male rats and overall data from female rats are underrepresented, studies regarding parental strains of the ZSF1 rat already indicate that one heterozygous mutant allele might impact cardiac health.

Nguyen et al.^8^ showed enhanced systolic blood pressure in lean male ZSF1 rats, as early as 12 weeks of age. In our developmental study on lean female ZSF1 rats of gradually increasing age, we observed a slight rise in blood pressure with age but not to a comparable degree.^12^ This indicates not only a sex‐specific variance but also the probability that the effects we report in female rats might be more pronounced in age‐matched male rats. Our previous study with multicentrically sampled lean rat data showed that sex may also impact parameters of myocardial functionality and NT‐proBNP levels.^6^ Compared to lean ZSF1 rats at <25 weeks of age, we found LVEF to be slightly decreased, but still within an healthy range, in all lean animals aged >25 weeks, independent of genotype and sex.^6^ Taken together, our results suggest that while the genotype of lean ZSF1 rats does not appear to directly influence myocardial parameters, advanced age and sex may do so.^6^

## 5 CONCLUSION

In summary, the present data indicate the onset of structural alterations in the heart and vasculature of female ZSF1 rats at 32 weeks of age, which, however, are not yet associated with functional impairments. Therefore, if sex‐ and age‐related restrictions are considered, the young lean ZSF1 rat remains a reliable control in ZSF1 based research. However, since the suitability of obese ZSF1 rats as an HFpEF model has been confirmed in multiple studies, it remains a question whether a lean controlgroup can be dispensed with entirely, thereby aligning preclinical study design more closely with clinical practice, where comparisons are typically made between treatment and placebo. Possibly an unusual idea in the context of preclinical research but maybe one worth considering.

## AUTHOR CONTRIBUTIONS

**Antje Schauer:** Conceptualization; data curation; formal analysis; validation; writing – original draft. **Beatrice Vahle:** Data curation; formal analysis. **Volker Adams:** Conceptualization; data curation; formal analysis; supervision. **Axel Linke:** Project administration; supervision. **Antje Augstein:** Data curation; formal analysis; methodology; validation; visualization; writing – review and editing.

## FUNDING INFORMATION

This research received no specific grant from any funding agency in the public, commercial, or not‐for‐profit sectors.

## CONFLICT OF INTEREST STATEMENT

A.L. reports grants from Novartis, personal fees from Medtronic, Abbott, Edwards Lifesciences, Boston Scientific, AstraZeneca, Novartis, Pfizer, Abiomed, Bayer, Boehringer, and other from Picardia, Transverse Medical, Claret Medical, outside the submitted work. The other authors report no conflicts.

## ETHICS STATEMENT

The study was approved by the Landesdirektion Sachsen (Approval numbers: TVV34/2020 and TVV42/2020) and carried out according to the institutional Animal Care guidelines as regulated by the German Federal law governing animal welfare.

## CLINICAL TRIAL REGISTRATION

This is not a clinical trial and thus was not registered.

## PATIENT CONSENT STATEMENT

Not applicable, as no identifiable human data were collected.

## PERMISSION TO REPRODUCE MATERIAL FROM OTHER SOURCES

No previously published material was reproduced in this manuscript.

## Data Availability

The data generated and/or analyzed during this study are available from the corresponding author upon reasonable request.
